# Cripto-1 as a Potential Target of Cancer Stem Cells for Immunotherapy

**DOI:** 10.3390/cancers13102491

**Published:** 2021-05-20

**Authors:** Hiroko Ishii, Said M. Afify, Ghmkin Hassan, David S. Salomon, Masaharu Seno

**Affiliations:** 1 GSP Enterprise, Inc., 1-4-38 12F Minato-machi, Naniwa-ku, Osaka 556-0017, Japan; ishii-gsp@tomoikebio.com; 2 Laboratory of Nano-Biotechnology, Graduate School of Interdisciplinary Science and Engineering in Health Systems, Okayama University, Okayama 700-8530, Japan; saidafify@s.okayama-u.ac.jp (S.M.A.); pthz2c4o@s.okayama-u.ac.jp (G.H.); 3 Division of Biochemistry, Chemistry Department, Faculty of Science, Menoufia University, Shebin ElKoum Menoufia 32511, Egypt; 4 Mouse Cancer Genetics Program, Center for Cancer Research, National Cancer Institute, Frederick, MD 21702, USA; salomond@mail.nih.gov

**Keywords:** Cripto-1, TGF-β, cancer stem cells, immunotherapy, antibody

## Abstract

**Simple Summary:**

Cancer immunotherapy is gaining attention as a potential fourth treatment following surgery, chemotherapy, and radiation therapy. Cancer stem cells have recently been recognized and validated as a key target for cancer treatment. Cripto-1, which is a GPI-anchored membrane-bound protein that functions as a co-receptor of Nodal, is a marker of cancer stem cells. Since Nodal is a member of the TGF-β family, which performs an important role in stem cells and cancer stem cells, the inhibition of Cripto-1 could be a strategy by which to block Nodal signaling and thereby suppress cancer stem cells. We propose that Cripto-1 may be a novel target for cancer immunotherapy.

**Abstract:**

The immune system has been found to be suppressed in cancer patients. Cancer cells are extremely resistant to chemotherapeutic drugs, conventional immunotherapy, or cancer antigen vaccine therapy. Cancer immunotherapy, which is mainly based on immune checkpoint inhibitors, such as those for PD-1, PD-L1, and CTLA4, is an effective treatment method. However, no immunotherapeutic target has been found that retains validity in the face of tumor diversity. The transforming growth factor (TGF)-β cytokine family possesses broad biological activity and is involved in the induction and/or transdifferentiation of helper T cells, which are important in immunotherapy. Nodal is a member of the TGF-β family playing important roles in tissue stem cells and cancer stem cells (CSCs), interacting with the co-receptor Cripto-1, as well as with Activin type IB (Alk4) and Activin typeIIreceptors, and maintaining stemness and Notch and Wnt/β-catenin signaling in CSCs. In recent years, it has been reported that Cripto-1 could be a potential therapeutic target in CSCs. Here, we review the accumulated literature on the molecular mechanisms by which Cripto-1 functions in CSCs and discuss the potential of Cripto-1 as an immunotherapeutic target in CSCs.

## 1. Introduction

Since van der Bruggen et al. first identified a human melanoma antigen recognized by cytolytic T cells in 1991 [1], tumor immunogenicity has been studied by many researchers as a potential feature to target in cancer treatment [2]. Tumor immunogenicity is responsible for the immune responses against cancer, activated as a host defense mechanism through antibody production and cell-mediated immunity. However, tumor immunogenicity varies depending on the type of cancer. Immunogenicity can be altered by mutations in tumors, which may in turn function as immunostimulants to induce cytokines or interferons. In this case, the concept of cancer vaccines is valid and has been developed in the past to establish cancer immunotherapy. The initial strategies were aimed at boosting the immune system that attacks cancer, but the therapeutic effect was not satisfactory on every patient. To accomplish a more robust response, research in cancer immunotherapy has been focused on T cell activation. T cells can attack cancer antigens that are presented on the cell surface. Immune checkpoint inhibitors (ICIs), such as programmed cell death 1 (PD-1) [3,4], programmed death-ligand 1 (PD-L1) [5,6], and cytotoxic T-lymphocyte antigen 4 (CTLA-4) [2,7,8], have been developed as effective agents in cancer immunotherapy. To enhance T cell activation, ICIs inhibit the interaction of T cells and cancer cells mediated by PD-1 and PD-L1, which enables cancer cells to escape from T-cell attack; therefore, ICIs’ mechanism of action is different from that of conventional cancer immunotherapy that directly boosts immunity as a method by which to attack cancer cells. Upon binding of PD-L1 to PD-1, signaling is transduced in T cells involving SHP2 to inhibit the proliferation and differentiation of T cells previously activated by a tumor antigen presented by antigen-presenting cells (APCs) through MHC. Therefore, ICIs allow activated T cells to recognize and attack tumor cells [9,10]. The successful therapeutic effect of ICIs is controversial, because only around 5–30% of all cancer patients who receive this therapy effectively respond to it. This response is not as good as that to conventional anticancer drugs, including monoclonal antibodies that target cancer-specific antigens. Therefore, other types of cancer immunotherapies are being developed [11].

The transforming growth factor β (TGF-β) family is a cytokine family with broad biological activity, involved in the induction of proliferation and/or differentiation of cells. Helper T cells, which are important in immunotherapy, are transdifferentiated by TGF-β, while TGF-β inhibits the differentiation of cytotoxic T lymphocytes (CTLs). TGF-β promotes the development of peripheral tumor-resident regulatory T (pTreg), Th17, Th9, and T follicular helper (Tfh) cells [12]. Cancer growth is regulated by various cytokines, including TGF-β, that are produced in the tumor microenvironment [13]. In recent years, Tauriello et al. reported that inhibition of TGF-β causes a strong and persistent cytotoxic T cell response against tumor cells, which prevents metastasis. They showed that increased TGF-β in the tumor microenvironment is a major mechanism of immune avoidance by promoting T cell elimination and blocking the acquisition of the T helper 1 (Th1) effector phenotype in advanced colorectal cancer [14]. Mariathasan et al. reported that enhanced TGF-β signaling in patients’ fibroblasts reduced patients’ responses to treatment in a cohort study analyzing specimens from patients with metastatic urothelial cancer. These authors also showed that the therapeutic co-administration of anti-TGF-β antibody and anti-PD-L1 antibody reduced TGF-β signaling in stromal cells and promoted T cell penetration into the center of the tumor [15].

Cripto-1 is a developmental oncoprotein highly expressed in many cancer cells, where it promotes tumorigenesis and is essential in embryogenesis. Cripto-1 is known as a co-receptor for Nodal, a member of the TGF-β family [16,17]. Cripto-1 is necessary for Nodal to bind to the Alk4 activin type I receptor. Several studies have shown that Cripto-1 plays an important role in early embryonic development as a co-receptor for Nodal [18,19]. Recently, Cripto-1 has been shown to be involved in the maintenance of cancer stem cells (CSCs) in various cancers [20,21,22,23,24,25].

In this review, we focus on Cripto-1 as a marker of CSCs associated with metastasis. The molecular function of Cripto-1 in CSCs is discussed together with the potential of Cripto-1 as a target for CSC immunotherapy.

## 2. Cripto-1 as a Novel Target for Immunotherapy

CSCs are present in most tumors. CSCs are characterized by their ability for self-renewal and differentiation, which maintains their original phenotype and, at the same time, allows the production of more differentiated cells within a tumor. CSCs are dormant and resistant to drug and radiation treatments, which leads to tumor recurrence and metastasis [26,27,28,29]. From this perspective, it is important to target CSCs in cancer therapy.

TGF-β has been shown to be an effective target in immunotherapy for cancer treatment. For example, two recent studies have found that TGF-β suppresses the antitumor immune response by inhibiting the infiltration of T cells into tumors [14,15]. On the other hand, Treg cells, which contribute to the inhibition of antitumor immune responses, were shown to harbor a latent form of TGF-β on the cell surface [30]. TGF-β has multiple functions in CSCs and can either inhibit or maintain various CSC traits [31]. TGF-β suppresses tumorigenesis by promoting the differentiation of CSCs, downregulating the expression of ABCG2, which acts as a transmembrane transporter, inducing chemoresistance in CSCs, and decreasing the aldehyde dehydrogenase 1 (ALDH1)-positive population, which is capable of self-renewal, as well as of enhancing tumor initiation/progression through CSCs. On the other hand, TGF-β promotes the stemness of CSCs in malignancy, induces the self-renewal of CSCs, and negatively regulates DNA methyltransferases, enhancing tumorigenesis. Oshimori et al. found that TGF-β in squamous cell carcinoma cells can significantly enhance glutathione metabolism and reduce the effectiveness of anticancer treatments by transcriptionally activating p21, which stabilizes NRF2 [32]. TGF-β can be a positive or negative regulator of cancer immunity [33]. Therefore, other TGF-β-related cytokines may be more convenient targets for immunotherapy.

Cripto-1 is a unique co-receptor required for Nodal activity. Blocking Cripto-1 would be a potential approach to inhibit Nodal, which is expressed in a number of different cancers [34,35] and which can enhance tumor growth and metastasis. Cripto is anchored by a glycosylphosphatidylinositol (GPI) motif to the surface of undifferentiated cells, including CSCs [36]. Cripto-1 contains EGF and CFC motifs which bind to Nodal and Alk4, respectively. Cripto-1 may be a suitable target for monoclonal antibody and vaccine therapies. Ligtenberg et al. and Witt et al. demonstrated that Cripto-1 vaccination was effective in inducing protective immune responses against metastases produced by sphere-forming CSCs of melanoma and mammary carcinoma cells [37,38]. In particular, in the study on melanoma, vaccination elicited a cytotoxic CD8^+^ T cell response. Another research group investigated the effect of Cripto-1 overexpression on macrophage activity and the underlying mechanism [39]. This study suggested that Cripto-1 enhances macrophage phagocytic activity and upregulates the production of anti-inflammatory and proinflammatory cytokines via the NF-κB signaling pathway. Stifter et al. investigated the effect of transporter-mediated tumor antigen expression associated with antigen processing on PD-1/ PD-L1-CD8 T cell priming using pancreatic ductal adenocarcinoma cells (PDACCs) in mice deficient for PD-1/PD-L1-competence [40]. They identified Cripto-1 as one of the two antigens that were expressed in the endoplasmic reticulum of PDACCs.

Cripto-1 has been shown to be highly expressed not only in embryonic stem cells but also in tumors where it promotes tumorigenesis [17,22], suggesting that it promotes the maintenance of a stem cell phenotype. Cripto-1 is involved in breast cancer tumorigenesis and is beginning to attract attention as a novel target for the treatment and diagnosis of breast cancer [41]. The simultaneous targeting of Nodal and Cripto-1 proteins has been shown to have therapeutic potential for oral squamous cell carcinoma [42]. Suppression of Cripto-1 expression inhibits cell stemness and EMT-related gene expression, significantly reducing self-renewal capacity, tumorigenesis, and metastasis in esophageal squamous cell carcinomas (ESCCs). Cripto-1 has also been demonstrated to regulate the Wnt/β-catenin signaling cascade and promote cell proliferation, migration, and invasion in hepatocellular carcinoma (HCC) [23]. Cripto-1 enhanced self-renewal capacity and conferred chemoresistance in HCC cells. Alowaidi et al. investigated the effect of Cripto-1 on pathways that control glioblastoma (GBM) cell function using phospho-specific protein microarray analysis, which suggested that angiogenesis could be mediated by Cripto-1 and that Cripto-1 might regulate the motility and infiltration of cancer cells [43,44]. Thus, Cripto-1 has the potential to be useful as a predictive and diagnostic marker in a therapeutic context (Table 1).

## 3. CSCs as a Therapeutic Target

CSCs are characterized as cells that divide asymmetrically and give rise to cells that produce a heterogeneous phenotype in tumors, providing resistance to chemo- and radiation therapy. The current medical therapies may be effective in destroying the majority of more differentiated cells in a tumor but not the small CSC subpopulation [53,54,55]. Additionally, CSCs are believed to play a critical role in the onset of metastasis. This may be due to the process of epithelial mesenchymal transition (EMT), which contributes to the generation of CSCs and metastasis [56,57]. Although the markers and driver pathways of CSCs vary among tumor types, the general stem cell characteristics, and the mechanisms of resistance to therapy appear common. CSCs show the expression of stemness markers, including Nanog, Oct4, and Sox2; display metabolic profiles distinct from those of terminally differentiated tumor cells; and reside in hypoxic microenvironments that sustain their long-term maintenance [58,59,60]. Targeting the mechanisms of chemoresistance in CSCs has produced some successful results in vitro during the last couple of years. Combinations of conventional drugs have been analyzed with the aim of targeting stemness markers, such as NANOG and STAT3, in CSCs [61,62]. The Wnt/β-catenin, Notch, hedgehog, and JAK–STAT pathways, which are considered essential for the maintenance of CSCs, are promising candidates for targeted therapy [63]. For instance, downregulation of transcriptional factors, such as STAT3, β-catenin, and Nanog, is considered a potential clinical target [64]. Not only cytoplasmic molecules but also cell surface markers, such as CD24, CD44, CD133, c-KIT, as well as Cripto-1, are conceivable as CSC targets. Monoclonal antibodies targeting CSC surface markers could also be effective to stop cancer recurrence when combined with surgery, chemotherapy, or radiotherapy. In this context, immune checkpoint inhibitors, which block the binding of PD-1 to PD-L1 or CTLA4 to B7, could be a critical therapeutic tool for targeting CSCs expressing PD-L1. Combinations of monoclonal antibodies targeting immune checkpoints and specific cell surface markers on CSCs could be more effective than single antibodies.

Specific microenvironments in niches are required for CSCs to self-renew and differentiate [65]. This concept of the existence of niches for stem cells was first formulated by Schofield, who proposed that a stem cell niche is essential to regulate stem cell fate and also demonstrated that stem cell behavior is influenced by the relationship between the cells within the niche [66,67]. The tumor microenvironment contains several components, including tumor-associated macrophages (TAMs), cancer-associated fibroblasts (CAFs), tumor-associated adipocytes (TAAs), tumor-associated dendritic cells (TADCs), vascular endothelial cells, and multipotent stromal cells (MSCs). Simultaneously, these cells secrete factors, including cytokines and growth factors. In addition, this microenvironment contains extracellular matrix (ECM) components and extracellular vesicles and is mostly hypoxic [68].

Stromal cells contribute more than 80% of the tumor volume in breast and pancreatic tumors and play a significant role in the development and maintenance of cancer [69]. The tumor stroma is thought to help the initiation and maintenance of CSCs, stimulate EMT, and enhance tumor progression, invasion, and recolonization [70]. Taking these properties into consideration, the tumor microenvironment (TME) may express various therapeutic targets that could be exploited to eliminate CSCs, even though practical ones are currently not available. Many cell populations within the TME comprise stem-like subpopulations that may share some common stem markers. Ravindran et al. recently reviewed the interactions between CSCs/cancer initiating cells (CICs) and TME [71]. CSC-specific therapies have long been proposed in conjunction with traditional chemotherapeutic regimens to kill both differentiated and undifferentiated cancer cell populations, thereby ensuring high therapeutic efficacy and preventing tumor recurrence. Overcoming the limitations of the current treatments and implementing innovative therapeutic strategies will provide more options to prolong patient survival.

## 4. Prospect of Antibody Drugs in Immunotherapy

**Figure 1:**
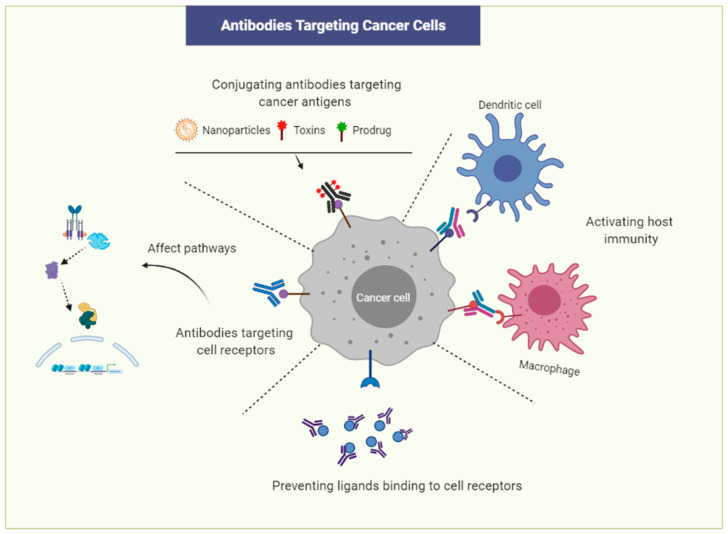
Strategies for targeting cancer cells with monoclonal antibodies (Mab). Mabs can be conjugated with toxins or nanoparticles that deliver drugs and toxic materials to cancer cells. Mabs could also target cancer cell receptors and inhibit cell signaling pathways responsible for cell growth, stem cell maintenance, and metastasis. Antibodies could bind ligands or receptors to prevent their interaction. Finally, antibodies could recruit different immune cells and activate host immunity against cancer cells.

Targeting cancer cells using antibodies can be one of the most effective strategies in cancer treatment (Figure 1). Numerous monoclonal antibodies (mAbs) have been developed since Genentech began to develop them against cancers [72]. Antibodies can induce tumor cell toxicity by the recruitment of immune cells, such as natural killer T cells and macrophages. The immune cells recognize the tumor-bound antibodies and perform phagocytosis or direct cytolysis, known as antigen-dependent cell-mediated cytotoxicity (ADCC). For instance, Trastuzumab, known as Herceptin, and Pertuzumab, known as Perjeta, which are monoclonal antibodies (mAbs) against human epidermal growth factor type 2 (Her2), are used to treat metastatic breast cancer overexpressing Her2 [73]. mAbs recognizing cancer cell-specific surface receptors may not only label tumor cells for immune destruction but also inhibit the downstream signaling pathways involved in regulating tumor cell proliferation and survival. Bevacizumab is a humanized mAb against vascular endothelial growth factor A (VEGF-A). Bevacizumab binds to VEGF-A, inhibits its binding to the VEGF receptor, and subsequently reduces tumor angiogenesis and restricts the blood supply to tumor cells [74]. Catumaxomab is a bispecific artificial antibody binding to both epithelial cell adhesion molecule EpCAM, a cancer stem cell antigen, and CD3, resulting in the activation of immune cells [75]. Catumaxomab kills EpCAM-positive cancer cells in malignant ascites and epithelial carcinomas. Cancer-specific antibodies can be conjugated to toxins or cytotoxic drugs and administered to target tumor cells or CSCs.

The presentation of tumor antigens by dendritic cells (DCs) can induce antitumor effects mediated by the host immune cells. DCs induce host immunity against tumor cells, by taking up tumor antigens and presenting them on major histocompatibility complex 1 (MHCI) molecules, thus resulting in the activation of cytotoxic T lymphocyte (CTL) [76]. On the other hand, PD-L1 is expressed by many tumors, such as kidney, melanoma, and lung cancers. PD-L1 stimulates PD-1 to suppress T cell receptors, resulting in the attenuation of the antitumor response of immune cells; in addition, the expression of PD-L1 was linked to tumor aggressiveness. This is one of the mechanisms by which cancer cells escape from immune cells, the so-called immune checkpoint escape. A number of antibodies against PD-1 and PD-L1 have been used as the choice of treatment of various cancers to block the PD-1 pathway and improve the immune response against tumor cells. CTLA-4 is another receptor with inhibitory roles regulating T cell activities. This receptor is also targeted by immune-modulatory strategies for treating different types of cancers in the same way as PD-1 [70,77].

This is an overall summary of immunotherapy, indicating the importance of the choice of antigens that are specific to cancer cells. Since Cripto-1 is overexpressed by various types of tumor cells [16,17,37,38], it might be a promising cancer-specific antigen. Adkins et al. demonstrated that monoclonal antibodies against the CFC domain of Cripto-1 interrupted Cripto-1/Nodal signaling and resulted in growth suppression of both colon and testicular cancer xenografts. Their data suggested that Cripto-1 signaling may be a novel target for the development of cancer therapeutics [78]. Kelly et al. described BIIB015, which is a conjugate of anti-Cripto antibodies and maytansinoid [79]. Bianco et al. showed that Cripto-1 performs an essential role in angiogenesis, which could be significantly blocked by antibodies [44]. Xing et al. also reported that mAbs against Cripto-1 inhibited tumor development and tumor growth of colon cancer xenografts. This report demonstrated that Cripto-1 mAbs induced cancer cell apoptosis that blocked Akt phosphorylation [80]. Anti-Cripto-1 mAbs also induced a mitochondrial cell death pathway, leading to leukemia cell growth inhibition and overcoming multidrug resistance [81]. Thus, Cripto-1 has been suggested as an attractive and novel target for the therapy of cancers overexpressing it. We established a novel artificially humanized Cripto-1 antibody [82] and are currently evaluating its ability to suppress tumor growth in vivo. Targeting Cripto-1 with mAbs or vaccines [37,38] should be an effective cancer treatment strategy.

Chimeric antigen receptor-modified T (CAR-T) may be the most advanced treatment method targeting CSCs. CSC-specific antigens, such as CD44, CD133, and epithelial cell adhesion molecule (EpCAM), have been considered as potential targets of CAR-T therapies. CAR-T recognizes cancer cells or CSCs and can eliminate xenograft tumors in vivo [83,84]. However, this strategy presents various problems, such as the inability to maintain CAR-T cells in a patient’s body for a long period of time, the extremely high cost of the treatment, and its low effectiveness in solid cancer. Therefore, the development of antibody-based drugs specific to CSCs would be advantageous. In addition, CAR-T cell-based therapies may inadvertently induce a cytokine storm in cancer patients, which can be lethal. As we mentioned in the second section, several studies have suggested a function for Cripto-1 in CSC.

## 5. Functions of Cripto-1 in CSCs

Abnormal signaling pathways can promote a variety of diseases, including cancer. In the development of cancer therapeutic agents, potential target molecules, including components of intracellular signaling pathways, are important, as exemplified by receptor tyrosine kinase inhibitiors (TKIs). Cripto-1 or teratocarcinoma-derived growth factor-1 (TDGF-1) was first identified and isolated as a cDNA in undifferentiated human and mouse teratocarcinoma cells [85]. Human Cripto-1 is a member of the epidermal growth factor (EGF)–Cripto-1/FRL-1/Cryptic (CFC) protein family. This oncofetal protein has emerged as a potential tumor biomarker [86,87,88,89,90,91,92,93]. The expression of Cripto-1 has been observed in different types of cancers, such as breast, colorectal, gastric, ovarian, lung, nonseminomatous testicular tumors, pancreatic ductal adenocarcinomas, basal cell carcinomas, and bladder carcinoma [16,17,92,93]. Cripto-1 is generally absent or present at very low levels in normal adult tissues [94], with the possible exception of tissue stem cell compartments in the breast, colon, and bone marrow, where it may be expressed at intermediate levels. Cripto-1 is involved in the stimulation of cell proliferation and migration and in angiogenesis.

### 5.1. Nodal/ALK4,7/Smad2 Signaling Pathway

In recent years, many studies have demonstrated that the Nodal/ALK4,7/Smad2 pathway is activated in malignant tumors, such as melanoma, breast cancer, endometrial carcinoma, and prostate cancer, and its activation is directly related to an increase in tumor cell plasticity [95,96,97]. Being involved in embryonic development as well as in CSC maintenance, Cripto-1 participates in this signaling pathway as a GPI-anchored membrane-bound coreceptor of Nodal in cis or as a soluble protein in trans.

**Figure 2:**
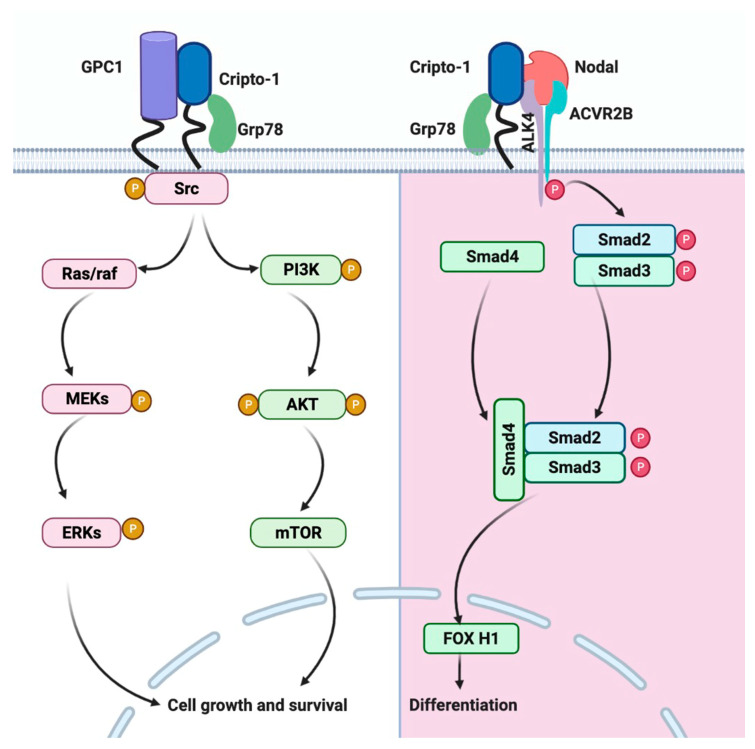
Signaling pathways that are activated by Cripto-1. Left: Cripto-1 Nodal-independent signaling via Glypican-1 activating c-Src/MAPK/AKT downstream signaling. Right: Canonical Nodal/ALK4,7/Smad2 signaling with Cripto-1 and Grp78 as co-receptor.

The canonical Nodal pathway is maintained by the interaction of Cripto-1, through its EGF domain, with Nodal and, through the CFC domain, with Alk4 or Alk7 [98,99,100,101]. This interaction leads to the transactivation of the ActRIIB receptor, which then phosphorylates the activin typeI receptors Alk4 or Alk7. The interaction of Cripto-1 through the CFC domain with Alk4/Alk7 can be enhanced by the heat shock protein Grp78, which forms a tertiary complex with Cripto-1 and Alk4/Alk7 [102]. Thus, a direct interaction between Cripto-1 and ALK4 is critical for the binding of Nodal to the ALK4/ActRIIB receptor complex, resulting in Smad2 phosphorylation [99,100,103]. In addition, Cripto-1 can interact with ALK7, modulating Nodal stimulation [98]. Activation of the ActRI/ActRIIB complex could in turn phosphorylate cytoplasmic Smad2 and/or Smad3, leading to their interaction with Smad4 and resulting in the activation of the Smad pathway [104,105]. The transcriptional complexes later initiate downstream gene transcription in conjunction with other transcription factors, such as FOXH1, which can promote breast cancer cell growth and invasion, and the Wnt/β-catenin pathway [106]. The Nodal/ALK4,7/Smad2 pathway involving Cripto-1 is presented in Figure 2.

### 5.2. Glypican-1/c-Src/MAPK/AKT Signaling Pathway

The interaction of Cripto-1 with the GPI-anchored protein glypican-1, membrane-associated heparan sulfate proteoglycan (HSPG), occurs in lipid raft membrane microdomains, where other signaling proteins, such as c-Src, are located [107]. Tyrosine kinase c-Src is activated, which is followed by the activation of mitogen-activated protein kinase (MAPK) and AKT in a Nodal/Smad-independent manner. The activation of Ras/Raf/MAPK and phosphoinositide 3-kinase (PI3K)/AKT signaling pathways by Cripto-1 affects cell growth, differentiation, and survival [108,109,110,111,112].

Since MAPK and AKT signaling pathways are known to stimulate cell proliferation and survival, Cripto-1 could be involved in the progression of human cancer through the abnormal activation of these two signaling pathways, independently of Nodal and ALK4. ERKs are upstream activators of MAPK. Furthermore, the phosphorylation of ERKs by MEKs enables MAPKs to translocate to the nucleus and activate transcription factors [113]. The AKT or protein kinase B (PKB) signaling cascade can also respond to the activation of receptor tyrosine kinases (RTK), such as ErbB4 [108,109]; this further enhances PI3K activity to produce phosphatidylinositol 3,4,5 triphosphates (PIP3), which binds to AKT phosphorylated by phosphoinositol-dependent kinase PDK1. Activated AKT dissociates from the plasma membrane to participate in a series of signaling events, which promote cell survival and proliferation. The glypican-1/c-Src/MAPK/AKT signaling pathway activated by Cripto-1 is presented in Figure 2.

When considering the signaling pathways induced by Cripto-1, it appears that antibodies targeting Cripto-1 could be suitable candidates for suppressing the proliferation and survival of CSCs.

## 6. Role of Cripto-1 in Metastasis and Recurrence

The invasion and metastasis of cancer cells into other organs are the most problematic issues in cancer treatment. In fact, approximately 90% of cancer deaths are due to distant metastases [114,115]. For the development of effective cancer treatments, therapies suppressing cancer invasion and metastasis must be achieved. Hu et al. analyzed whole-exome sequence data from 136 primary and metastatic breast, colon, and lung cancers [116]. A driver mutation, which controls the spread of cancer cells but is associated with drug resistance, is frequently found in metastatic cancers. Even after the surgical removal of the primary tumor, a small number of cancer cells may remain and subsequently contribute to the recurrence of a drug-resistant tumor, due to the survival of CSCs [115,117]. Clinical tests are being developed, with the aim of detecting subclinical and microtumors early. Approaches that therapeutically attack drug-resistant CSCs will be necessary to improve patient survival.

Cripto-1 has been demonstrated to mechanistically contribute to metastasis, recurrence, and drug resistance [23,46,118,119]. Cripto-1 is a promising immunotherapeutic target in hepatocellular carcinoma, glioma, melanoma, prostate cancer, and metastatic breast cancer [37,38,47,52,120,121].

Karkampouna et al. assessed the combination of sorafenib and doxorubicin with Cripto-1 pathway inhibitors in xenografts from patients overexpressing Cripto-1 and showed that sorafenib resistance could be avoided by the inhibition of the Cripto-1 pathway [52]. They also reported that the stable overexpression of Cripto-1 in human HepG2 cells caused epithelial–mesenchymal transition, increased the expression of cancer stem cell markers, and enhanced cell proliferation and migration and drug resistance. Focà et al. developed a monoclonal antibody that targets the CFC domain of Cripto-1, similar to the antibody that was developed by Biogen [78,120]. They reported that this antibody prevented the activation of c-Src and ERK in melanoma C8161 cells expressing Cripto-1. They also suggested that this antibody could neutralize the oncogenic activity of Cripto-1 and overcome drug resistance in cancer cells and tissues that express Cripto-1 [120]. Witt et al. studied a model of vaccination with DNA encoding mouse Cripto-1, targeting metastatic breast cancer and CSCs [38]. In this model, metastasis of breast cancer cells from two different transgenic models was inhibited, demonstrating that a Cripto-1 vaccine could reduce the burden of lung metastases. Watanabe et al. separated cell populations with high and low Cripto-1 expression from NTERA2/D1 embryonal carcinoma cells [121]. Using these two cell populations, they showed that the population with high levels of Cripto-1 expressed stem cell markers that were regulators of pluripotent embryonic stem cells. Alowaidi et al. showed that Cripto-1 expression in invasive glioblastoma enhanced the expression of vimentin and twist, which contribute to the migration and invasion of cancer cells [42]. Their data suggested that Cripto-1 was useful as a predictive and diagnostic marker. Furthermore, Zoni et al. found that the expression of Cripto-1 in metastases of prostate cancer (PCa) patients correlated with a decrease in survival rate [118]. They also found that Cripto-1 knockdown decreased bone metastases in a preclinical mouse model of PCa.

**Figure 3:**
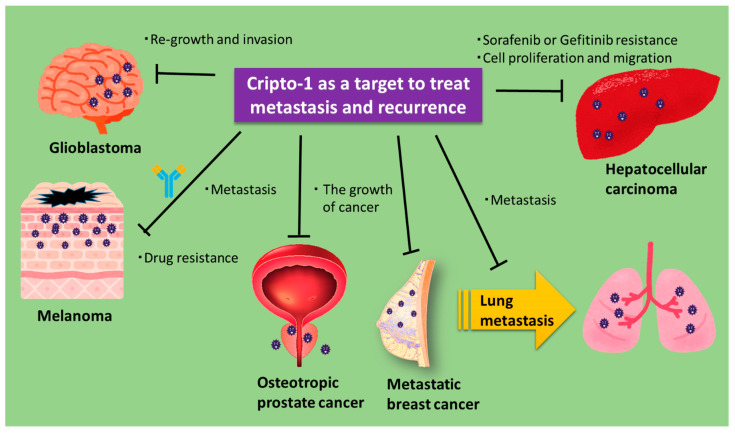
Antagonizing Cripto-1 can decrease metastasis and tumor recurrence. A summary of the tumor-suppressing effect of Cripto-1 antagonists on metastasis and recurrence of tumors.

These results suggest that Cripto-1 is an effective target against cancer metastasis and recurrence in many types of cancer (Figure 3).

## 7. Conclusions

While many cancer researchers are focusing on immunotherapy as a promising therapeutic strategy for cancer, targets more effective than immune checkpoint molecules should be explored. The development of new therapies targeting cancer stem cells, which could be efficacious for treating cancer, should be considered. Cripto-1 is a potential target for cancer immunotherapy. As we summarized in this paper, the therapeutic effect of targeting Cripto-1 in metastatic cancer and CSCs has recently been demonstrated in different cancers. Antibodies or vaccines targeting Cripto-1 have been experimentally and clinically validated.

## Figures and Tables

**Table 1:** Recent research papers on Cripto-1 as a therapeutic target.

Organ	Cancer Cells	CSCs
Breast	Regulation of human Cripto-1 expression by nuclear receptors and DNA promoter methylation in human embryonal and breast cancer cells [45]	Cripto-1 Plasmid DNA Vaccination Targets Metastasis and Cancer Stem Cells in Murine Mammary Carcinoma [38]
	Cripto-1 as a novel therapeutic target for triple-negative breast cancer [46]	
Brain	Cripto-1 overexpression in U87 glioblastoma cells activates MAPK, focal adhesion, and ErbB pathways [43]	
	Investigating the role of CRIPTO-1 (TDGF-1) in glioblastoma multiforme U87 cell line [47]	
	Cripto-1 localizes to dynamic and shed filopodia associated with cellular migration in glioblastoma cells [48]	
The others	Overexpression levels of cripto-1 predict poor prognosis in patients with prostate cancer following radical prostatectomy [49]	Cripto-1 acts as a functional marker of cancer stem-like cells and predicts prognosis of the patients in esophageal squamous cell carcinoma [25]
	Expression and functional role of CRIPTO-1 in cutaneous melanoma [50]	Cripto-1 contributes to stemness in hepatocellular carcinoma by stabilizing Dishevelled-3 and activating Wnt/β-catenin pathway [23]
	The role of Nodal and Cripto-1 in human oral squamous cell carcinoma [42]	Exogenous Cripto-1 Suppresses Self-Renewal of Cancer Stem Cell Model [51]
	CRIPTO promotes an aggressive tumor phenotype and resistance to treatment in hepatocellular carcinoma [52]	Dynamic regulation of the cancer stem cell compartment by Cripto-1 in colorectal cancer [24]
